# The Surgical Diseases of the Tongue

**Published:** 1861-06

**Authors:** 


The following communication from the London Lancet was presented by Mr. Weeden Cooke to the Harveian Society. The subject is highly interesting, and one to which the attention of the dentist is sometimes called.—[Ed.

The Surgical Diseases of the Tongue.—Casually glancing only at the varying aspects of the papillæ in diseases which come more immediately under the notice of the physician, Mr. Cooke took a rapid survey of those injuries and diseases of the structure of the tongue which are generally considered to be surgical cases. *Wounds*, whether incised, contused, or lacerated, invariably healed rapidly in healthy constitutions—a circumstance which was doubtless due to the high organization of the part, and in some measure, perhaps, to the beneficial influence of the salivary secretion. *Idiopathic glossitis* was a remarkable disease, arising at times without any assignable cause, and happily entirely under the control of the surgeon. Incisions and antiphlogistic treatment very quickly overcame the malady. *Hypertrophy*, depending either on chronic inflammatory action or on the tuberculosis, was a more serious and protracted lesion of this organ. In either case it was an indication of diminished vital power, and for its relief cod-liver oil and iron, with or without oxide of potassium, and generous diet, generally proved most beneficial; but it was a troublesome affection, the cure of which required much patience and prudence on the part of the sufferer. Sea air was always most serviceable in these cases. *Ulceration* occurring in this organ was rather aphthous, dyspeptic, strumous, syphilitic, or cancerous. The proper diagnosis of the origin of the ulcer was important, inasmuch as the treatment required in each case was peculiar to itself, and could not be beneficial unless addressed to the systemic condition out of which arose the ulcer under consideration.

After describing the apthous, dyspeptic, and strumous ulcers of the tongue, and exhibiting drawings of them, Mr. Cooke contrasted especially the syphilitic and cancerous ulcers of this organ, and stated that they were often confounded,—that syphilitic ulcers with much ulceration of the tongue had been treated as cancerous, remaining for years without benefit, when, the proper diagnosis being arrived at, they had been cured so rapidly as to excite wonder on the part of the patient. Whenever a person presented himself with an ulcer of the tongue, it was always necessary to investigate minutely his whole constitutional history, observing with especial care any cutaneous eruption of even the most trifling character. Thus armed, we may take into consideration the peculiarities of the local ulcer, and so arrive at a mature judgment respecting the group in which it should be classified and the treatment it should undergo. "And in these investigations," said Mr. Cooke, "it is necessary to take nothing for granted. The patient often deceives himself, or is really ignorant of his having a syphilitic taint; and if we do not examine carefully for ourselves, we also shall be led astray in the diagnosis upon which depend the life and happiness of the patient, as well as our own credit as skillful surgeons." After dividing the different characteristics of syphilitic and cancerous ulcers of the tongue, Mr. Cooke alluded shortly to the distinctions between *syphilitic and cancerous tumors* of this organ, showing that the soft elastic feel of the one contrasted vividly with the hard cartilaginous sensation produced by the other. *Operations* for cancer of the tongue never succeeded in checking the disease for any length of time. If it did not return in the organ itself, the sublingual or submaxillary glands soon took on the same action, and the misery of the patient was enhanced by the transfer. Of the three modes of operating—by ligature, by the knife, and by the ecraseur—Mr. Cooke had no doubt that the knife was the most desirable. He had seen very bad results from the ecraseur. *Local and constitutional treatment* was of the utmost value in the malignant, as in the non-malignant affections of the tongue. In the latter class a cure was always to be effected; while in the former, much relief from suffering was to be obtained, and in some instances the disease had been arrested in its progress. Lotions of chlorate of potash, sulphate of copper, and borax were recommended, together with the internal administration of the mineral acids, bark, iron, and cod-liver oil.

